# Supplementary material for: Clinical Insights into Non-Alcoholic Fatty Liver Disease and the Therapeutic Potential of Flavonoids: An Update
Source: Nutrients. 2025 Mar 9;17(6):956. doi: 10.3390/nu17060956 (PMC11944923; doi:10.3390/nu17060956)
Supplement: Supplementary file 1 [file nutrients-17-00956-s001.zip › nutrients-3517368-supplementary.pdf]

Supplementary Table S1. The summary of (A) clinical trials and (B) meta-analyses in NAFLD treatment.

A. Clinical trials in NAFLD treatment

Study (year)	Study Design and Comparator (if applicable)	Number of participants	Drug/Substance	Dosage [mg/d]	Condition	Treatment/ use duration	Endpoints/Measures	Outcomes	Limitations
Ferro (2022) [1]	a randomized, double-blinded, controlled trial	94	Bergamot polyphenol fraction and *Cynara Cardunculus* extract	300mg/day	hepatic steatosis	six weeks	• serum uric acid (SUA),<br>• lipids,<br>• glucose,<br>• anthropometric parameters	• change in SUA, especially in participants with moderate/severe hepatic steatosis<br>• in those with the highest baseline SUA (>5.4 mg/dL) a greater reduction compared to the lowest baseline SUA (-7.8% vs. +4.9%; adjusted p = 0.04)	the use of ultrasound for diagnosing NAFLD
Ferro (2020) [2]	a randomized, double-blinded, controlled trial	102	Bergamot polyphenol fraction and *Cynara Cardunculus* extract	300mg/day	hepatic steatosis	twelve weeks	• liver fat content by transient elastography	• reduction in liver fat content (−48.2 ± 39 vs. −26.9 ± 43 dB/m, p = 0.02)<br>• CAP reduction in	CAP score might not be proper to evaluate steatosis stages in detail

							• serum transaminases, lipids, and glucose	patients aged over 50, with android obesity, overweight/obesity as well as in women	
Ghanbari (2024) [3]	a randomized clinical trial study	50	grape seed extract	250 mg of proanthocyanidins	NAFLD patients	two months	• oxidative stress markers (TAC, MDA, SOD, GPx, CAT, and IL-6)<br>• quality of life (QoL)	• reductions in IL-6 and MDA<br>• increases in TAC, SOD, and GPx levels<br>• improvement in physical limitations, general health, and total physical health	socioeconomic status, social support, lifestyle factors, and comorbidities that were not assessed could impact participants' QOL scores
Li (2024) [4]	a randomized, double-blind, placebo-controlled crossover clinical trial	36	quercetin	500 mg	patients with NAFLD	12 weeks	• intrahepatic lipid content	• decreased the intrahepatic lipid contents from 11.5% ± 6.4% to 9.6% ± 5.8%<br>• body weight and body mass index were mildly reduced	slightly larger body weight loss in the quercetin group
Naeini (2022) [5]	a pilot double-blind, placebo-controlled,	44	naringenin	200 mg	overweight/obese patients with NAFLD	4 weeks	• cardiovascular risk factors	• reduction in the atherogenic index of plasma value, serum non-	small study sample size Short duration

	randomized clinical trial							HDL-C levels, total cholesterol/HDL-C, triglyceride/HDL-C, low-density lipoprotein cholesterol/HDL-C, and non-HDL-C/HDL-C ratios<br>• reduction in BMI and visceral fat level<br>• increase in the mean corpuscular hemoglobin concentration	
Namkhah (2021) [6]	placebo-controlled, parallel randomized, double-blind clinical trial study	44	naringenin	200 mg	overweight/obese patients with NAFLD	4 weeks	• lipid profile,<br>• aminotransferase levels,<br>• the severity of steatosis, as well as the probability of fibrosis	• reduced TG, TC, and LDL-c<br>• increased HDL-c<br>• reduced percentages of NAFLD grades<br>• no significant changes in AST, ALT and NFS	small study sample size short duration
Neshatbini (2024) a	a randomized	50	soy isoflavone	100 mg	patients with NAFLD	12 weeks	• metabolic status	• reduced serum TG,	small sample size

[7]	placebo controlled clinical trial							• LDL-c, and TC<br>• no changes in HDL-C, BP, and glycemic parameters	
Neshat-bini (2024) b [8]	a randomized placebo-controlled clinical trial	50	soy isoflavone	100 mg	patients with NAFLD	12 weeks	• the level of fibroblast growth factor-21 and fetuin-A	• decrease in ALT, AST, CAP score, steatosis grade, and an increase in the level of fetuin-A<br>• fibrosis grade and serum levels of GGT and FGF-21	small sample size
Notarnicola (2024) [9]	a randomized clinical trial	62	Orange (hesperidin)	400g	patients with MASLD	4 weeks	• liver function and steatosis	• liver steatosis decreased in the treatment group by 30%<br>• no significant changes in fibrosis or plasma liver enzymes	the absence of liver biopsies for histopathological and mechanistic characterization
Vilar-Gomez (2021) [10]	a prospective study	452	Dietary intake		patients with NAFLD, and the human patatin-like phospholipase domain-containing 3-gene		• fibrosis severity	• higher isoflavones (mg/day) intake was inversely associated with an increased risk of significant fibrosis	underestimation of macronutrient intakes

								(stage of fibrosis ≥2)	
Yang (2024) [11]	a clinical randomized controlled trial	20	epigallocatechin gallate	300 mg	patients with NAFLD	24 weeks	• the effectiveness of EGCG in the treatment of NAFLD	• improved hepatic fat content<br>• changes in both the waist circumference and waist-to-hip ratio<br>• drop in this serum DPP4 levels	small sample size
Yari (2021) [12]	an open-labeled randomized controlled trial	100	flaxseed and hesperidin (combination)	30g 1000mg	patients with NAFLD	12 weeks	• the clinical effects of flaxseed and hesperidin in patients with NAFLD	• decreases in plasma levels of alanine aminotransferase, indices of insulin resistance and insulin sensitivity, fasting glucose, and fatty liver index	no placebo has been known for flaxseed
Zhu (2022) [13]	a case-control and an intervention study	74	anthocyanins	160 mg/day	NAFLD patients	12 weeks	• anti-inflammatory therapies in NAFLD	• Decrease levels of caspase-1, IL-1b, and IL-18	result reflect patients' systemic inflammation status rather than hepatic inflammation status

B. Meta-analyses in NAFLD treatment

Study (year)	Study Design and Comparator	Number of trials (subjects)	Drug/Substance	Dosage [mg/d]	Condition	Treatment/ use duration	Endpoints/Measures	Outcomes	Limitations
Kalopitas (2021) [14]	meta-analysis of prospective cohort studies	8 studies (622 individuals)	silymarin	140 to 2100 mg. The majority of the trials used 280 mg of silymarin	subject with NAFLD, obese or overweight	8 to 48 weeks	• ALT and AST values<br>• changes in BMI<br>• changes in liver histology	• reduction in the levels of transaminases, irrespective of weight loss.	NAFLD cohort of people from a Western country (the United States)
Li (2023) [15]	meta-analysis of prospective cohort studies	12 (831 patients with NAFLD, with 418 participants in the intervention group and 413 participants in the control group)	Flavonoids (hesperidin, Silybum, anthocyanin, genistein, dihydromyricetin)	94 to 2100 mg d−1	adults with NAFLD	8 to 48 weeks	• liver function: ALT, AST,γ-glutamyl transpeptidase GGT, CK-18M30, fibrosis score, steatosis score,<br>• lipid profile TG, LDL-c, HDL-c, TC,<br>• inflammation: TNF-α, hs-CRP, [NF-κB],<br>• insulin resistance	• beneficial effects of flavonoids on:<br>• ALT (SMD = −3.59, p =<br>• 0.034),<br>• AST (SMD= −4.47, p = 0.001),<br>• GGT (SMD =−8.70, p = 0.000),<br>• CK-18M30 (SMD =−0.35, p = 0.042),<br>• TG (SMD =−0.37, p = 0.001),<br>• LDL-C (SMD =−0.38, p = 0.039),<br>• TC (MD= −0.25 mmol/l, p = 0.017),	differences in study design (flavonoid type, dosage, intervention duration, and ethnicity)

								• steatosis score (MD =−18.97, p = 0.30),<br>• TNF-α (MD= −0.88, p = 0.000),<br>• NF-κB (MD= −1.62, p = 0.001).	
Li (2024) [16]	meta-analysis of randomized controlled trials	26 trials (2375 subjects)	silymarin	n/a	adults with NAFLD	2-12 weeks in most included studies	• liver damage	• reduction in TC, TG, LDL-c, HOMA-IR<br>• increases in HDL-c<br>• decreased levels of ALT, AST, fatty liver index, fatty liver score	The types, the doses of silymarin, and the lifestyle management of the patients were different among the included RCTs.
Liu (2024) [17]	meta-analysis of randomized controlled trials	37 trials (2509 subjects)	silymarin, artichoke leaf extract, berberine, catechins, and naringenin		adults with NAFLD	4 to 48 weeks	• therapeutic effect on NAFLD	• artichoke leaf extract confers reduces AST and ALT levels, and LDL-c<br>• Naringenin reduces TG, TC levels and improve HDL-c<br>• catechins significantly reduced BMI levels	compared the effects of different natural products

Mahmoodi (2020) [18]	meta-analysis of randomized controlled trials	15 trials	green tea or green tea catechin	384 mg to 1080 mg	healthy individuals and people with nonalcoholic fatty liver disease	3 to 48 weeks	• liver enzymes	• reduction of the levels of liver enzymes in participants with NAFLD<br>• in healthy subjects, a small significant increase in liver enzymes	relatively heterogeneous population
Yang (2022) [19]	meta-analysis of randomized controlled trials	46 trials (2,173 participants)	naringenin, anthocyanin, hesperidin, catechin, silymarin, genistein	200 mg<br>n/a<br><br>1000mg<br>550mg<br>140-700mg<br>250 mg	adults with NAFLD	4 to 48 weeks	• the effectiveness of dietary flavonoids in the treatment of NAFLD	• naringenin decreased the percentage of NAFLD grade, TG, TC, and LDL-c and increased HDL-c<br>• hesperidin decreased BMI, AST, ALT, TG, TC, HOMA-IR<br>• catechin decreased BMI, HOMA-IR, and TG<br>• silymarin was effective in improving ALT and AST and reducing hepatic fat	heterogeneity in some outcomes

								accumulation and liver stiffness	
Zhang (2024) [20]	meta-analysis of randomized controlled trials	16 trials (1135 subjects)	Chinese medicine combined with Silibinin	n/a	adults with NAFLD	n/a	• The efficacy and quality assessment of traditional Chinese medicine in combination with Silibinin	• the total effective rate of the combined treatment group was significantly higher than that of Silibinin alone<br>• improving levels of ALT, AST, TC, TG, and GGT	traditional Chinese medicine prescription and medication lacked standardization

AST— aspartate aminotransferase, ALT—alanine aminotransferase , BMI— body mass index, BP— blood pressure, CAT— catalase, CAP— controlled attenuation parameter, CK-18M30— cytokeratin 18-M30, DPP4— dipeptidyl peptidase 4, EGCG— epigallocatechin gallate, FGF-21— fibroblast growth factor 21, GGT— gamma-glutamyl transferase, GPx— glutathione peroxidase, HDL-c— high-density lipoprotein cholesterol, HOMA-IR— homeostasis model assessment-insulin resistance, hs-CRP— high-sensitive C-reactive protein, ICAM-1— intercellular adhesion molecule 1, IL— interleukin, MASLD— metabolic dysfunction-associated steatotic liver disease, LDL-c— low-density lipoprotein cholesterol, MDA— malondialdehyde, n/a—not available or not applicable, NAFLD— non-alcoholic fatty liver disease, NF-κBb— nuclear factor-κB, NFS— non-alcoholic fatty liver score, SOD— superoxide dismutase, SUA—serum uric acid, TAC— total antioxidant capacity, TC— total cholesterol, TG— triglycerides, TNF-α— tumor necrosis factor-alpha, QoL— quality of life

1. Ferro, Y., et al., *Citrus Bergamia and Cynara Cardunculus Reduce Serum Uric Acid in Individuals with Non-Alcoholic Fatty Liver Disease.* Medicina (Kaunas), 2022. **58**(12).
2. Ferro, Y., et al., *Randomized Clinical Trial: Bergamot Citrus and Wild Cardoon Reduce Liver Steatosis and Body Weight in Non-diabetic Individuals Aged Over 50 Years.* Front Endocrinol (Lausanne), 2020. **11**: p. 494.
3. Ghanbari, P., et al., *Grape seed extract supplementation in non-alcoholic fatty liver disease.* Int J Vitam Nutr Res, 2024. **94**(5-6): p. 365-376.
4. Li, N., et al., *Quercetin intervention reduced hepatic fat deposition in patients with nonalcoholic fatty liver disease: a randomized, double-blind, placebo-controlled crossover clinical trial.* Am J Clin Nutr, 2024. **120**(3): p. 507-517.

5. Naeini, F., et al., *Effects of naringenin supplementation on cardiovascular risk factors in overweight/obese patients with nonalcoholic fatty liver disease: a pilot double-blind, placebo-controlled, randomized clinical trial.* Eur J Gastroenterol Hepatol, 2022. **34**(3): p. 345-353.
6. Namkhah, Z., et al., *Does naringenin supplementation improve lipid profile, severity of hepatic steatosis and probability of liver fibrosis in overweight/obese patients with NAFLD? A randomised, double-blind, placebo-controlled, clinical trial.* Int J Clin Pract, 2021. **75**(11): p. e14852.
7. Neshatbini Tehrani, A., et al., *The effect of soy isoflavones supplementation on metabolic status in patients with non-alcoholic fatty liver disease: a randomized placebo controlled clinical trial.* BMC Public Health, 2024. **24**(1): p. 1362.
8. Neshatbini Tehrani, A., et al., *The effect of soy isoflavones on non-alcoholic fatty liver disease and the level of fibroblast growth factor-21 and fetuin A.* Sci Rep, 2024. **14**(1): p. 5134.
9. Notarnicola, M., et al., *Daily Orange Consumption Reduces Hepatic Steatosis Prevalence in Patients with Metabolic Dysfunction-Associated Steatotic Liver Disease: Exploratory Outcomes of a Randomized Clinical Trial.* Nutrients, 2024. **16**(18).
10. Vilar-Gomez, E., et al., *Impact of the Association Between PNPLA3 Genetic Variation and Dietary Intake on the Risk of Significant Fibrosis in Patients With NAFLD.* Am J Gastroenterol, 2021. **116**(5): p. 994-1006.
11. Yang, M., et al., *Epigallocatechin gallate alleviates non-alcoholic fatty liver disease through the inhibition of the expression and activity of Dipeptide kinase 4.* Clin Nutr, 2024. **43**(8): p. 1769-1780.
12. Yari, Z., et al., *The efficacy of flaxseed and hesperidin on non-alcoholic fatty liver disease: an open-labeled randomized controlled trial.* Eur J Clin Nutr, 2021. **75**(1): p. 99-111.
13. Zhu, X., et al., *Upregulated NLRP3 inflammasome activation is attenuated by anthocyanins in patients with nonalcoholic fatty liver disease: A case-control and an intervention study.* Clin Res Hepatol Gastroenterol, 2022. **46**(4): p. 101843.
14. Kalopitas, G., et al., *Impact of Silymarin in individuals with nonalcoholic fatty liver disease: A systematic review and meta-analysis.* Nutrition, 2021. **83**: p. 111092.
15. Li, L., et al., *Does Flavonoid Supplementation Alleviate Non-Alcoholic Fatty Liver Disease? A Systematic Review and Meta-Analysis of Randomized Controlled Trials.* Mol Nutr Food Res, 2023. **67**(23): p. e2300480.
16. Li, S., et al., *Administration of silymarin in NAFLD/NASH: A systematic review and meta-analysis.* Ann Hepatol, 2024. **29**(2): p. 101174.
17. Liu, H., et al., *Effects of different natural products in patients with non-alcoholic fatty liver disease-A network meta-analysis of randomized controlled trials.* Phytother Res, 2024. **38**(7): p. 3801-3824.
18. Mahmoodi, M., et al., *Effects of green tea or green tea catechin on liver enzymes in healthy individuals and people with nonalcoholic fatty liver disease: A systematic review and meta-analysis of randomized clinical trials.* Phytother Res, 2020. **34**(7): p. 1587-1598.
19. Yang, K., et al., *Efficacy and safety of dietary polyphenol supplementation in the treatment of non-alcoholic fatty liver disease: A systematic review and meta-analysis.* Front Immunol, 2022. **13**: p. 949746.

20. Zhang, X., et al., *Efficacy of traditional Chinese medicine combined with Silibinin on nonalcoholic fatty liver disease: A meta-analysis and systematic review.* Medicine (Baltimore), 2024. **103**(5): p. e37052.
